# Telehealth With Comprehensive Live-Fed Real-World Data as a Patient Care Platform for Lung Cancer: Implementation and Evaluation Study

**DOI:** 10.2196/45331

**Published:** 2024-06-05

**Authors:** Di Zheng, Yanhong Shang, Jian Ni, Ling Peng, Xiaoming Tan, Zhaoxia Dai, Yizhuo Zhao, Aiqin Gu, Jiying Wang, Yanyan Song, Xiaofeng Li, Junping Zhang, Wei Heng, Cuiying Zhang, Chunling Liu, Hui Li, Yingying Du, Jianfang Xu, Dan Wu, Xuwei Cai, Rui Meng, Xiaorong Dong, Yaoping Ruan, Liyan Jiang

**Affiliations:** 1 Department of Medical Oncology Shanghai Pulmonary Hospital Tongji University School of Medicine Shanghai China; 2 Department of Medical Oncology Affiliated Hospital of Hebei University Baoding China; 3 Department of Respiratory Disease Zhejiang Provincial People's Hospital Affiliated People's Hospital, Hangzhou Medical College Hangzhou China; 4 Department of Respiratory and Critical Care Medicine Renji Hospital Shanghai Jiao Tong University School of Medicine Shanghai China; 5 Department of Thoracic Medical Oncology II The Second Hospital of Dalian Medical University Dalian China; 6 Department of Respiratory and Critical Care Medicine Shanghai Chest Hospital Shanghai Jiao Tong University School of Medicine Shanghai China; 7 Department of Biostatistics Clinical Research Institute Shanghai Jiao Tong University School of Medicine Shanghai China; 8 Internal Medicine of Thoracic Oncology Baotou Tumor Hospital Baotou China; 9 Department of Thoracic Oncology The Affiliated Bethune Hospital of Shaanxi Medical University Taiyuan China; 10 Department of Medicine, Respiratory, Emergency and Intensive Care Medicine The First Affiliated Hospital of Soochow University Suzhou China; 11 Cancer Center Inner Mongolia Autonomous Region People's Hospital Huhehot China; 12 The Second Ward Department of Pulmonary Medicine Xinjiang Medical University Affiliated Tumor Hospital Urumqi China; 13 The First Affiliated Hospital of Anhui Medical University Hefei China; 14 Department of Thoracic Surgery Cixi People's Hospital Ningbo China; 15 Cancer Center Union Hospital, Tongji Medical College Huazhong University of Science and Technology Wuhan China; 16 College of Mathematics and Computer Science Zhejiang A&F University Hangzhou China; 17 Metafame Technologies Inc Shanghai China

**Keywords:** telehealth, real-world data, patient engagement, lung carcinoma, patient-reported outcomes

## Abstract

**Background:**

Telehealth has emerged as a popular channel for providing outpatient services in many countries. However, the majority of telehealth systems focus on operational functions and offer only a sectional patient journey at most. Experiences with incorporating longitudinal real-world medical record data into telehealth are valuable but have not been widely shared. The feasibility and usability of such a telehealth platform, with comprehensive, real-world data via a live feed, for cancer patient care are yet to be studied.

**Objective:**

The primary purpose of this study is to understand the feasibility and usability of cancer patient care using a telehealth platform with longitudinal, real-world data via a live feed as a supplement to hospital electronic medical record systems specifically from physician’s perspective.

**Methods:**

A telehealth platform was constructed and launched for both physicians and patients. Real-world data were collected and curated using a comprehensive data model. Physician activities on the platform were recorded as system logs and analyzed. In February 2023, a survey was conducted among the platform’s registered physicians to assess the specific areas of patient care and to quantify their before and after experiences, including the number of patients managed, time spent, dropout rate, visit rate, and follow-up data. Descriptive and inferential statistical analyses were performed on the data sets.

**Results:**

Over a period of 15 months, 16,035 unique users (13,888 patients, 1539 friends and family members, and 174 physician groups with 608 individuals) registered on the platform. More than 382,000 messages including text, reminders, and pictures were generated by physicians when communicating with patients. The survey was completed by 78 group leaders (45% of the 174 physician groups). Of the participants, 84% (65.6/78; SD 8.7) reported a positive experience, with efficient communication, remote supervision, quicker response to questions, adverse event prevention, more complete follow-up data, patient risk reduction, cross-organization collaboration, and a reduction in in-person visits. The majority of the participants (59/78, 76% to 76/78, 97.4%) estimated improvements in time spent, number of patients managed, the drop-off rate, and access to medical history, with the average ranging from 57% to 105%. When compared with prior platforms, responses from physicians indicated better experiences in terms of time spent, the drop-off rate, and medical history, while the number of patients managed did not significantly change.

**Conclusions:**

This study suggests that a telehealth platform, equipped with comprehensive, real-world data via a live feed, is feasible and effective for cancer patient care. It enhances inpatient management by improving time efficiencies, reducing drop-off rates, and providing easy access to medical history. Moreover, it fosters a positive experience in physician-patient interactions.

## Introduction

According to the GLOBOCAN 2020 report, the cancer mortality rate is higher in China than that in developed countries [1]. Lung cancer remains the most common and deadliest type of cancer, with an estimated 0.82 million new cases and 0.72 million deaths in 2020 in China [1]. In contrast to the rapidly declining mortality rate for lung cancer in high-income countries between 2000 and 2012 [2], the trend in the lung cancer mortality rate was stable in China from 2000 to 2016 [3]. Despite favorable survival outcome data for Chinese patients in international randomized clinical trials, these data do not reflect the real-world situation for the general population. The less-than-optimal progress in cancer control, especially in terms of the mortality rate, may be attributed to health care disparities between different regions, particularly urban and rural areas [4,5]. Clinical trial data from inadequately represented cancer patient populations could be complemented with real-world evidence to better inform health care practice and policy decisions [6].

The rapid development and adoption of new treatment regimens have made posttreatment care a critical factor in extending the cancer survival rate and improving patients’ quality of life [7]. Concurrently, telehealth has quickly become a major care delivery mechanism in recent years, a trend accelerated by the COVID-19 pandemic. One ongoing effort to sustain and scale digital health involves enabling data sharing and integration across different health systems [8]. Consequently, most telehealth systems today rely on point-in-time medical records that do not contain historical records nor data from other institutes. To overcome this data barrier, the platform implemented in this study has the capability to acquire medical records directly from patients.

Though there are perceivable benefits to having comprehensive medical records for telehealth, enabling comprehensive and longitudinal data for each patient involves tremendous effort. Such data are also critical for deriving conclusive real-world evidence [9]. Data acquisition must be inclusive, especially of vital signs related to the patient’s daily health status throughout the entire treatment period, in addition to diagnosis and treatment information [10]. Although this is currently achievable with the adoption of wearables and mobile devices, there are still tremendous challenges in longitudinally compiling patients’ journeys as there are no unified nationwide platforms that can consolidate all relevant data from all health care institutions in China [11,12]. The ever-increasing mobility of patients across the country has exacerbated the issue of data segmentation. Presumably, due to the recent improvement in annual income per household and the deployment of interstate health care systems, many patients opt for top-tier hospitals regardless of the travel distance from their home. It is quite common for one patient to receive treatment from different hospitals at various stages, while the hospital systems remain disconnected. The lack of longitudinal data from such fragmented health services may also contribute to subpar care and survival outcomes [13,14].

The distinct feature of telehealth, in which this study is interested, is its use as an adjunct to traditional physical visits and face-to-face consultations, particularly for posttreatment care and continuity of care from a physician’s perspective. Much of the research on telehealth usage has been focused on patients as the user population. Williams and Shang [15] examined telehealth use among a low-income, minority population in the United States and found the use of telehealth varies based on race, employment status, identified gender, education level, and age. Acoba et al [16] studied racial disparities in cancer patients during telehealth visits and confirmed that satisfaction with the visit is different between races. Turner et al [17] evaluated the experiences of health care providers and professionals during the COVID-19 pandemic and concluded the need for implementation strategies and necessary policies. Specifically for cancer patients, teledermatology has emerged as a popular mechanism [18]. In a cross-sectional study, Lama et al [19] found that more than one-half of cancer survivors use the internet or telehealth to access providers.

One of the specific aspects being assessed is follow-up, a unique challenge for cancer care in China, primarily due to the substantial patient-to-physician ratio [20]. Follow-ups using patient-reported outcomes (PROs) can improve the overall survival rate due to early relapse detection and better performance status at relapse. A study published in 2017 found that patients who reported their symptoms via an online tool survived 7 months longer than those who received usual care through regular screenings [21]. A previous meta-analysis of 21 studies also demonstrated that the reporting of PROs, including quality of life and disease symptoms, were significantly associated with tumor response to anticancer therapies such as chemotherapy, targeted therapy, and radiotherapy [22].

The platform used in this study, named WeDoc, is cloud-based and currently focuses on lung cancer. It consists of a mobile app for physicians, a WeChat mini-program for patients, and a cloud-based data and analytical component serving as the back end. The platform contains comprehensive, longitudinal medical records sourced from all relevant hospitals and supplemented with third-party test results, PROs, follow-up data, and more. The underlying data model is highly customizable to individual physicians’ needs and contains curated fields commonly used for cancer clinical research.

## Methods

### Overview

A cloud-based telehealth platform was built and launched for licensed oncologists and their patients. Patient medical records were collected and curated into a proprietary lung cancer data model. Physician and patient activities are recorded on the platform. A survey containing qualitative and quantitative questions was conducted 20 months after launch. Descriptive statistics and regression analysis were conducted on the survey data.

Analysis was conducted on 2 sets of data: activities recorded on the platform and results from a usage survey. Both sets of data were gathered from the perspective of physicians, as the goal in the first stage of this platform is to function as an assistant for physicians.

### Platform Implementation and Recording of User Activity

The back end of the platform features a data processing pipeline; data and process management interfaces; and cloud repositories for raw, curated, and research data. Original data are deidentified, masking all personal details. These data are then abstracted and reviewed by trained personnel, and the abstracted data are consolidated, checked for quality, and committed to the real-world data repository.

Patients are invited to the platform by their oncologists and can form a user group with family members or friends. Oncologists can invite physicians and caregivers to create a treatment group, facilitating remote collaboration and simplifying hospital transfers. Patient reminders, assessments, and symptom feedback are gathered, and any potential adverse events are escalated to the primary oncologist.

The system’s data model incorporates the schema of electronic medical records, patient outcome reports, and periodic progression assessments by physicians, with a primary focus on lung cancer data. Data abstraction and data quality assurance involve both manual processes and regularly executed algorithms.

### Survey Design and Questionnaire

The platform records the number of registered users and their activities. In March 2023, about 20 months after launch, an online usage survey was carried out using a WeChat survey mini-program. The program was pushed to all registered users as a study advertisement. The survey consisted of both qualitative and quantitative questions. Instead of individual physicians, each treatment group leader was asked to compile the group’s experience and provide responses. This approach was taken because the group leader dictates the use of the platform, and each group member may only utilize a subset of its functions.

The survey questions were designed to evaluate physicians’ patient care experiences using the platform. This includes basic functions and follow-up, their estimation of promptness in answering patient questions, patient risk reduction, cross-organization collaboration, and handling out-of-town patients. Quantitative questions asked for the number of both outpatients and inpatients managed, reduction in the number of physical visits, patient drop-off rates, and time spent collecting medical history during each visit. All identifiable information about participants was removed, and each individual was assigned a unique participant ID. 

**Table 1:** Survey question categories, descriptions, and answer options.

Question category and description					Answer options
**Patient care functions**					
	A1: (Efficient communication) The platform serves as a communication channel for physicians to provide online notification of important matters.				Single-choice selection of binary options (agree or disagree) for each statement as a checkbox selection
	A2: (Remote supervision) The platform enables physicians to provide continuous supervision and remote interaction.				Single-choice selection of binary options (agree or disagree) for each statement as a checkbox selection
	A3: (Medical history retrieval) The platform offers patients’ medical history and communication records for physicians to review.				Single-choice selection of binary options (agree or disagree) for each statement as a checkbox selection
	A4: (Patient administrative processes) The platform helps hospital appointment scheduling for both outpatient and inpatient procedures.				Single-choice selection of binary options (agree or disagree) for each statement as a checkbox selection
	A5: (Response to patient question on time) The platform enables physicians to promptly answer patients’ questions without in-person visits.				Single-choice selection of binary options (agree or disagree) for each statement as a checkbox selection
	A6: (Adverse event prevention) The platform enables physicians to timely capture potential adverse reactions from patient feedback.				Single-choice selection of binary options (agree or disagree) for each statement as a checkbox selection
**Follow-up**					
	B1: (Treatment status availability) Before: It was hard to acquire patient status. After: Patient status is easy to gather from the platform.				Single-choice selection of binary options (agree or disagree) for each statement as a checkbox selection
	B2: (Survival status availability) Before: It was hard to acquire survival status. After: Survival status is provided on the platform.				Single-choice selection of binary options (agree or disagree) for each statement as a checkbox selection
	B3: (Data comprehensiveness) Before: Records were incomplete. After: Multidimensional, comprehensive data are available on the platform.				Single-choice selection of binary options (agree or disagree) for each statement as a checkbox selection
	B4: No differences between before and after using the platform.				Single-choice selection of binary options (agree or disagree) for each statement as a checkbox selection
**Response promptness**					
	C: With the platform, are you able to respond to patient inquiries quicker than before?				Single-choice selection of 3 options (yes, no, or unknown) for each question as a radio button selection
**Patient risk reduction**					
	D: After using the platform, do you feel that your patients have a lower risk of adverse reactions?				Single-choice selection of 3 options (yes, no, or unknown) for each question as a radio button selection
**Cross-organization collaboration**					
		E: Have you established collaborations across different departments, hospitals, or even regions through the platform?		Single-choice selection of 3 options (yes, no, or unknown) for each question as a radio button selection	
**Management of remote patients**					
	F: Is managing out-of-town patients more convenient for you by using the platform?				Single-choice selection of 3 options (yes, no, or unknown) for each question as a radio button selection
**More patients managed per unit time**					
	G: With the platform, how many more patients can you manage within the same amount of time?				Single-choice selection of 5 quantitative ranges: 10%-20%, 20%-50%, 50%-100%, >100%, 0%
**In-person visits saved**					
	H: After using the platform, what is your estimation of the average number of in-person visits reduced per patient per year?				Single-choice selection of 5 quantitative ranges: 1-3, 4-6, 7-10, >11, 0
**Prior telehealth experience**					
	I: Before using WeDoc, did you use any other telehealth platforms for patient management?				Single-choice selection of yes or no
**Patient management specifics**					
	**How many minutes per day do you spend managing patients?**				
			J1: Before	Quantitative values entered by participants	
			J2: After	Quantitative values entered by participants	
	**What is the total number of patients you manage?**				
			K1: Before	Quantitative values entered by participants	
			K2: After	Quantitative values entered by participants	
**Outpatient management**					
	**How many outpatient visits in total do your lung cancer patients have per month?**				
			L1: Before	Quantitative values entered by participants	
			L2: After	Quantitative values entered by participants	
	**What percentage of your lung cancer patients are likely to miss their outpatient visits each month?**				
			M1: Before	Quantitative values entered by participants	
			M2: After	Quantitative values entered by participants	
**Inpatient management**					
	**How many lung cancer patients do you see for inpatient treatment per month?**				
			N1: Before	Quantitative values entered by participants	
			N2: After	Quantitative values entered by participants	
	**What percentage of your inpatients discontinue their treatment each month?**				
			O1: Before	Quantitative values entered by participants	
			O2 – After	Quantitative values entered by participants	
**Medical history collection**					
	**How many minutes do you spend collecting the medical history in each patient visit?**				
			P1: Before	Quantitative values entered by participants	
			P2: After	Quantitative values entered by participants	

### Statistical Analysis

Descriptive statistics and regression analysis were conducted using the Python program. For descriptive analysis, we calculated the means, medians, standard deviations, and ranges. For quantitative questions regarding usage before and after, we used the Shapiro-Wilk test to assess the normal distribution of the data. Subsequently, we used the Wilcoxon rank sum test to evaluate the significance of the data sets. We used G*Power [23] to analyze the difference between 2 dependent means (matched pairs), setting the alpha at .05, beta at .2, and dz at 0.5. Assuming a medium-level difference between the before and after groups, a sample size of 27 was considered sufficient for the tests.

### Ethical Considerations

This study was reviewed and approved by Yinchuan Ningfei Internet Hospital (approval number HLWYJ-2022-016). Participants were not compensated for their participation.

## Results

### Activities Recorded on the Platform

Over a period of 15 months, 608 physicians from 153 hospitals registered on the platform. The hospitals were from 21 of the 34 total provinces in China. Of the physicians, 92.8% (142/153) were from hospitals rated as Grade III, Level A, which is the highest rating according to the latest statistics [24] (Table 2). From a departmental perspective, 46.3% (125/270) of the physicians were from the oncology department, 41.9% (113/270) were from the department of respiratory and critical care medicine, and 11.9% (32/270) were from other departments.

**Table 2:** Physician and patient profiles registered in the system, including the numbers of hospitals, departments, physicians, treatment groups, and patients.

Characteristics		Results
**Hospitals (n=153), n (%)**		
	Grade III, Level A	142 (92.8)
	Others	11 (7.2)
**Departments (n=270), n (%)**		
	Oncology	125 (46.3)
	Respiratory and critical care medicine	113 (41.9)
	Others	32 (11.9)
**Physicians (n=608), n (%)**		
	Treatment group leader	174 (28.6)
Treatment groups, n		211
Patients and family members, n		15,427
**Patients (n=13,888), n (%)**		
	Nonresident patients	7826 (56.3)

One of the platform’s features for physicians is creating treatment groups by including other physicians. Among the 608 physicians, 174 have one or more groups. There are a total of 211 groups, with most physicians managing between 1 and 3 groups. A patient may be part of multiple groups, depending on the group’s purpose and treatment stage. For instance, a patient undergoing inpatient chemotherapy might initially be in a group with a radiologist in the hospital but later transferred to a follow-up group consisting only of the lead oncologist and the follow-up assistant. Table 2 describes the profiles of physicians and treatment groups.

In addition to physicians and caregivers, there are 15,427 patients and family members on the platform. Within that user group, 9.98% (1539/15,427) are family members or friends.

Table 3 demonstrates the message types and quantities of physician-patient communication from the system activity logs. More than 382,000 messages including text messages, reminders, and pictures were recorded during the study period. Text was the most commonly used message type. Pictures and voice messages were used significantly less often than text messages. Reminders, patient education materials, team messages, and scaled assessments were usually initiated by physicians for different purposes.

**Table 3:** Activity log of the message types and quantities between physician-patient communication.

Message type	Typical usage	Message count, n
Text	Chats between patients and physicians	222,012
Reminder	Appointments and preparation items for appointments	66,985
Picture	Pictures in chat with patients	32,548
Patient education	General patient education through formats such as articles, videos, and URLs	27,538
Team message	Messages between physicians within the same group	19,779
Scaled assessment	Patient self-assessment of various aspects	8005
Voice	Voice messages for patients	5884

### Survey Questionnaire Responses

#### Participant Characteristics

A total of 78 group leaders participated in the survey, representing 44.8% (78/174) of the treatment groups. All the groups were associated with Group III, Level A hospitals. Participant characteristics including city locations, gender distribution, departments, age groups, and prior experience with telehealth platforms are summarized in Table 4.

**Table 4:** Profiles of participants in the survey questionnaire (N=78).

Characteristics			Results, n (%)
**City location**			
	Beijing, Shanghai, or Guangzhou	33 (42)	
	Others	45 (58)	
**Gender**			
	Female	36 (46)	
	Male	42 (54)	
**Departments **			
	Oncology	46 (59)	
	Respiratory	26 (33)	
	Others	6 (8)	
**Age group (years)**			
	20-30	4 (5)	
	30-40	15 (19)	
	40-50	33 (42)	
	50-60	24 (31)	
	>60	2 (3)	
**Prior telehealth usage**			
	No	25 (32)	
	Yes	53 (68)	

#### Qualitative Question Results

For questions A1 to F, which included the topics of communication efficiency, remote supervision, question response times, adverse event prevention, follow-up data completeness, patient risk reduction, cross-organization collaboration, and remote patient management, participants provided qualitative answers to each question. The results are shown in Table 5. A positive answer indicates agreement with the statement or yes to the question. A negative answer indicates disagreement with the statement or no to the question. Most of the questions received positive answers except for the topic of cross-organization collaboration, which had nearly neutral feedback: 54% positive versus 46% negative. The questions of treatment status availability (B1), survival status availability (B2), and data comprehensiveness (B3) contain both before and after statements. A negative answer may indicate that the participant only disagrees with part of the statement. Therefore, the final results of these questions indicated less favorable evaluations of the WeDoc tool.

**Table 5:** Results of the qualitative survey questions (N=78).

Question description		Survey results, n (%)
**A1: Efficient communication**		
	Positive	76 (97)
	Negative	2 (3)
**A2: Remote supervision**		
	Positive	73 (94)
	Negative	5 (6)
**A3: Medical history retrieval**		
	Positive	69 (89)
	Negative	9 (12)
**A4: Patient administrative processes**		
	Positive	58 (74)
	Negative	20 (26)
**A5: Respond to patient questions on time**		
	Positive	69 (89)
	Negative	9 (12)
**A6: Adverse event prevention**		
	Positive	67 (86)
	Negative	11 (14)
**B1: Treatment status availability**		
	Positive	68 (87)
	Negative	10 (13)
**B2: Survival status availability**		
	Positive	58 (74)
	Negative	20 (26)
**B3: Data comprehensiveness**		
	Positive	68 (87)
	Negative	10 (13)
**B4: No difference**		
	Positive	8 (10)
	Negative	70 (90)
**C: Response promptness**		
	Positive	70 (90)
	Negative	8 (10)
**D: Patient risk reduction**		
	Positive	70 (90)
	Negative	8 (10)
**E: Cross-organization collaboration**		
	Positive	42 (54)
	Negative	36 (46)
**F: Management of remote patients**		
	Positive	78 (100)
	Negative	0
**I: Prior telehealth experience**		
	Positive	53 (68)
	Negative	25 (32)
**G: Additional patients managed per unit time**		
	0%	6 (8)
	10%-20%	20 (26)
	20%-50%	28 (36)
	50%-100%	7 (9)
	>100%	17 (22)
**H: In-person visits saved per year**		
	0	6 (8)
	1-3	12 (15)
	4-6	30 (39)
	7-10	21 (27)
	>11	9 (12)

On the question of the number of patients managed using the tool, 36% (28/78) of the participants estimated that they were able to see 20%-50% more patients with the same amount of time spent. However, 6 of the participants estimated a 0% increase. Similarly, on the question regarding the number of in-person patient visits reduced, 39% (30/78) estimated 4 to 6 visits saved per year, and 6 respondents did not see a reduction.

Among the 78 participants, 53 (68%) had prior telehealth usage experience, while it was the first time using a telehealth tool for the rest of the group. We divided the participants into 2 groups, with and without telehealth usage experience, for some of the additional analyses.

#### Quantitative Question Results

For questions J to P, participants were asked to provide quantitative values for their experiences both before and after using the tool. Table 6 summarizes the values for each question. The “Unknown” category indicates null values in the survey, and these responses were omitted in the analysis. The highest number of unknown answers we received was for the question about the number of patients managed before using the tool. We used G*Power analysis for the remaining nonnull before-and-after pairs to ensure that there was a sufficient sample for analysis. With an assumption of medium differences between the before and after groups, at least 27 samples had to be present in the group.

**Table 6:** Results for the quantitative survey questions (N=78).

Question description			Unknown responses, n (%)		Valid responses									
					Minimum-maximum		Median		Mean (SD)		Mean improvement, %		*P* value for the before-after comparison^a^	
**Time spent managing patients (minutes)**														<.001
	J1: Before	18 (23)		10-180		30		50.5 (45.1)		N/A^b^				
	J2: After	10 (13)		3-120		20		25.5 (22.7)		88				
**Number of patients managed**														.01
	K1: Before	20 (26)		0-800		40		105.7 (177.4)		N/A				
	K2: After	13 (17)		1-1606		100		324.3 (428.8)		63				
**Monthly number of outpatient lung cancer patients**														.66
	L1: Before	8 (10)		0-2000		85		221.8 (352.2)		N/A				
	L2: After	8 (10)		0-2000		80		237.1 (369.6)		—^c^				
**Outpatient drop-off rate (%)**														<.001
	M1: Before	11 (14)		0-80		25		26.8 (21.1)		N/A				
	M2: After	11 (14)		0-50		10		13.1 (11.4)		105				
**Monthly number of lung cancer inpatients**														.59
	N1: Before	2 (3)		0-350		60		110.1 (93.5)		N/A				
	N2: After	2 (3)		0-350		70		116.2 (94.8)		—				
**Inpatient drop-off rate (%)**														<.001
	O1: Before	5 (6)		0-50		10		14.9 (12.1)		N/A				
	O2: After	5 (6)		0-100		5		9.2 (16.2)		62				
**Time collecting medical history (minutes)**														<.001
	P1: Before	5 (6)		2-180		10		14.0 (28.8)		N/A				
	P2: After	3 (4)		1-120		3		8.8 (19.2)		57				

^a^Assessed using Wilcoxon tests.

^b^N/A: not applicable.

^c^No improvement.

To better understand the differences between the before and after results, we used the Shapiro algorithm to test whether the values fell within a normal distribution. For normally distributed data series, a *t* test can be used to compare the pairs. Otherwise, the Wilcoxon test is a more suitable method. Since the *P* values of the Shapiro test were all <.001, which is much lower than the common hypothesis threshold of .05, we concluded that none of the pairs were normally distributed. Therefore, Wilcoxon tests were performed on the before-and-after pair data (Table 6). The Wilcoxon results suggest that there were 2 questions that were not significantly different between before and after the platform: the monthly number of outpatients admitted and the monthly number of inpatients admitted. This result is quite explainable, as the telehealth tool itself is not aimed at recruiting new patients; therefore, the monthly numbers of patients remained nearly the same. For the topics that had significant changes, we calculated the improvements based on the mean values collected in the survey, which are also shown in Table 6.

Although the survey was not specifically designed to compare the group with prior telehealth platform experience with the group without prior experience, we discovered that 68% (53/78) of the participants had used telehealth tools before. In order to understand the experience by group, we carried out a Wilcoxon test to compare the responses before and after (Table 7). The numbers of monthly admitted outpatient and inpatient lung cancer patients still did not change significantly. However, there was also no significant change in the number of patients managed, suggesting that physicians may not manage more patients using WeDoc than with other telehealth platforms.

**Table 7:** Results for the quantitative survey questions for those who had prior telehealth platform experience (n=53).

Question description		Unknown responses, n (%)	Valid responses					
			Minimum-maximum	Median	Mean (SD)	Mean improvement, %	*P* value for the before-after comparison^a^	
**Time spent managing patients (minutes)**								<.001
	J1: Before	5 (9)	10-180	30	50.7 (46.8)	N/A^b^		
	J2: After	5 (9)	3-120	20	28.6 (25.2)	77		
**Number of patients managed**								.06
	K1: Before	11 (21)	1-800	40	125.8 (199.5)	N/A		
	K2: After	6 (11)	1-1200	100	322.3 (403.4)	—^c^		
**Monthly number of outpatient lung cancer patients**								.75
	L1: Before	4 (8)	0-2000	60	214.7 (388.9)	N/A		
	L2: After	4 (8)	0-2000	60	225.6 (407.9)	—		
**Outpatient drop-off rate (%)**								<.001
	M1: Before	7 (13)	0-80	30	28.2 (22.7)	N/A		
	M2: After	7 (13)	0-50	10	13.9 (12.1)	102		
**Monthly number of lung cancer inpatients**								.65
	N1: Before	0	0-350	60	109.6 (100.0)	N/A		
	N2: After	0	0-350	60	113.8 (98.4)	—		
**Inpatient drop-off rate (%)**								<.001
	O1: Before	3 (6)	0-50	10	14.7 (13.3)	N/A		
	O2: After	3 (6)	0-12	5	5.7 (4.1)	159		
**Time collecting medical history (minutes)**								<.001
	P1: Before	3 (6)	2-180	8	15.3 (34.1)	N/A		
	P2: After	3 (6)	1-120	3	9.3 (22.8)	63		

^a^Assessed using Wilcoxon tests.

^b^N/A: not applicable.

^c^No improvement.

## Discussion

### Principal Findings

#### Specific Feedback About the Platform

Results from activity logs and survey responses demonstrate the feasibility of cancer patient care using telehealth with a live-transmitted real-world database. Specifically, 84% (65.6/78, SD 8.7) of participants responded positively to questions A1 through F. The lowest scores were for patient administrative processes and survival status. Patient administrative processes in China are complex and not the primary focus of this platform, while obtaining updated survival status during follow-up is clearly an area for improvement. Another area that did not stand out was cross-organization collaboration, presumably due to the deployment of other specialized platforms such as Multidisciplinary Team, which is popular in China. Of the participants, 92% believed that they could manage more patients with the same amount of time, and an equal number of physicians agreed that the platform saves at least one or more instances of in-person visits.

Our analysis of the before and after experiences of the same population showed that 5 of the 7 categories were significantly different after use of the platform, as determined using the Wilcoxon signed rank test. The 2 categories that were not significantly different were the monthly numbers of outpatient and inpatient admissions. These 2 factors are unaffected by the use of any patient management tool; thus, they are indeed irrelevant to our telehealth platform.

#### Perceptions of Those With Prior Telehealth Usage

Given that 68% of the participants had prior experience with telehealth platforms, analyzing this population alone yielded similar results, except that the number of patients managed did not meet our significance value assumption of .05. This implies that, although managing more patients is a benefit of telehealth platforms, it may not be unique to ours. The strengths of a telehealth platform with real-world data are manifested in the categories of time efficiency, drop-off rates, and access to patients’ medical histories.

#### Remote Patient Management

The adoption of remote patient management was evident in the patient profiles, which showed that more than one-half of patients, about 56.3%, were nonresidents; 941 patients had transferred from one hospital to another, and almost 1500 patients had prior diagnoses or treatments from hospitals other than their current hospital. Taking hospitals in Shanghai as an example, the platform showed that about 35% of patients were from cities other than Shanghai. Although more than one-half of the patients were from adjacent provinces such as Jiangsu and Zhejiang, some travel thousands of miles from places like Heilongjiang, Sichuan, and Liaoning. Because of the unbalanced health care situation in China, it is quite common for patients to be diagnosed in one hospital and receive treatments at another. Despite significant improvements over the past few decades, the best oncologists and medical facilities are still heavily concentrated in top cities.

#### Text as the Dominant Message Type

The activity log indicated that text was the most commonly used message type to communicate with patients. The use of pictures and voices messages was significantly lower than that of text. Reminders were also quite popular, followed by educational materials. The preliminary analysis did not reveal significant differences in usage patterns among physicians, so we did not present usage data by physician profile.

#### Security and Privacy

With the adoption of the Personal Information Protection Law (PIPL) [25] in China on November 01, 2021, all systems handling data from Chinese citizens must be compliant with the law. This law is widely seen as China’s equivalent of the EU General Data Protection Regulation (GDPR) [26]. The system in question acts as both a data handler and data processor. It controls the scope of data usage based on the level of consent obtained from users, making user consent a mandatory prerequisite for successful user registration. By separating raw data and identifiers from curated, deidentified data, the system ensures the proper implementation of data protection policies.

From an operational perspective, privacy protection remains one of the most significant challenges in building such a platform. The challenge is less technical, as there are rich sets of mechanisms available, such as data anonymization, encryption, access control, and audit. The main challenge comes from the perceptions and cooperation of patients. Ideally, patients and their relatives should also have access to real-world data, enabling them to participate in treatment decisions. Apart from patient perceptions, potential malpractice concerns also hinder data sharing, preventing people from gaining strategic insights. Health care policymakers and scientific researchers need to collaborate with data analysts to promote a proper data sharing process.

### Limitations

Although this study is based on a live system with real-world data and experiences, the findings remain preliminary. At present, the platform only provides services to the lung cancer population, and the results of this study are derived from physicians from a subset of the treatment paradigm. Although the user base of the platform encompasses both physicians and patients, future research involving a broader population, including more physicians and direct patient experiences, may yield new, insightful findings. It would also be interesting to expand to other diseases. Given the large quantity of chat messages accumulated on the platform, a detailed examination of these messages paired with language processing models would be a fascinating next step.

### Conclusion

This study demonstrates the feasibility of using telehealth for patient management. As the focus of cancer treatment shifts toward patient care, telehealth in the form of mobile apps, web-based interfaces, or other formats will play an increasingly critical role in enabling physicians to maintain close contact with patients, regardless of physical location. We advocate for the integration of telehealth with comprehensive real-world medical record data, so that such a platform can provide patient management capabilities. This could eventually lead to improved quality of life and survival rates of cancer patients.

## Abbreviations

GDPR: General Data Protection Regulation
PIPL: Personal Information Protection Law
PRO: patient-reported outcome

## Data Availability

The data sets analyzed during this study are available from the corresponding author on reasonable request.
